# Therapists’ Personal Identity Factors and Professional Self-Efficacy: A Path Analysis Model

**DOI:** 10.3390/bs16081425

**Published:** 2026-08-19

**Authors:** Alessio Gori, Eleonora Topino

**Affiliations:** 1 Department of Health Sciences, University of Florence, Via di San Salvi 12, Pad. 26, 50135 Florence, Italy; 2 Integrated Psychodynamic Psychotherapy Institute (IPPI), Via Ricasoli 32, 50122 Florence, Italy; 3 Department of Theoretical and Applied Sciences, e-Campus University, Via Isimbardi 10, 22060 Novedrate, Italy; eleonora.topino@gmail.com

**Keywords:** personal identity, professional identity, online therapy, occupational self-efficacy, therapist self-efficacy

## Abstract

The integration of the Internet into clinical practice has facilitated the implementation of digital mental health interventions, including eTherapy. In this context, the present study examined factors associated with Therapist Self-Efficacy during synchronous video-based eTherapy, specifically focusing on Self-Esteem, General Self-Efficacy, and Insight Orientation while controlling for Years of Clinical Practice. A sample of 290 mental health professionals (39% psychologists; 61% psychotherapists) completed a survey including the Therapist Self-Efficacy Scale, General Self-Efficacy Scale, Rosenberg Self-Esteem Scale, and Insight Orientation Scale. A path analysis was performed to analyse the collected data. Results showed a significant total mediation model. Specifically, significant and positive total effects in the relationships of Self-Esteem and General Self-Efficacy with Professional Self-Efficacy emerged. Furthermore, Insight Orientation fully mediated these associations. Years of Clinical Practice was not significantly associated with Therapist Self-Efficacy. These findings may inform the development of tailored eTherapy training for mental health professionals.

## 1. Introduction

Over recent decades, technological developments have enabled the implementation of eTherapy in mental health care. eTherapy has been defined as a “*professional therapeutic interaction that makes use of the Internet to connect qualified mental health professionals and their clients*” (Rochlen et al., 2004, p. 270). This modality has broadened access to specialized mental health services by reducing physical barriers (e.g., distance, difficulty in moving; Leykin et al., 2012; Stoll et al., 2020), and psychosocial barriers (e.g., perceived stigma; Marques et al., 2010). At the same time, the implementation of this modality requires the professional to adopt some safeguards so that the therapeutic process is not hindered both by technical (e.g., there must be a good connection and an adequate camera resolution; Waller et al., 2020) and/or ethical (e.g., Internet communications must be secure; Scharff, 2012; Stoll et al., 2020) issues. Indeed, synchronous video-based psychotherapy requires therapists to adapt established clinical skills to a setting characterized by physical distance, reduced access to bodily and contextual cues, possible technical disruptions, and different challenges in maintaining emotional connection and therapeutic presence. Research has shown that therapists may experience lower therapeutic presence during teletherapy and that concerns about connecting with clients online are associated with less favorable perceptions of this modality. Conversely, mastery of teletherapy-specific interventions has been associated with stronger working alliance, real relationship, and therapeutic presence (Békés et al., 2021; Békés et al., 2023; Aafjes-Van Doorn et al., 2024). In this framework, the dimension of professional self-efficacy appears to be of great relevance (Gori et al., 2022a): indeed, it is essential that clinicians who decide to practice eTherapy feel capable of adapting to this web-based modality while maintaining confidence in their professional skills. Professional self-efficacy refers to an individual’s confidence in their ability to achieve quality results in the professional tasks of their specific occupation (Fraser et al., 2018; Yoo & Cho, 2020). In the mental health field, it was associated with higher compassion satisfaction (Saleem & Hawamdeh, 2022), job and role satisfaction (Lent et al., 2009), lower levels of emotional exhaustion, depersonalization (Gunduz, 2012), and anxiety (Daniels & Larson, 2001), as well as more realistic definitions of clinical goals and more effective performance (Reese et al., 2009). Considering these meaningful outcomes, the study of the personal factors associated with mental health therapists’ professional self-efficacy is of great interest both in the clinical and research fields. Previous evidence has defined Professional Self-Efficacy as a component of professional identity (Heled & Davidovitch, 2021; Tan et al., 2017), which refers to the meanings, values, roles, knowledge, and competencies through which individuals define themselves as members of a profession (Tan et al., 2017; Wiles, 2013). Experienced counsellors have described their personal characteristics and life experiences as informing who they are professionally (Alves & Gazzola, 2011), while research on counsellor and therapist development has conceptualized professional growth as a lifelong process of integration between the personal and professional selves, supported by continuous reflection (Rønnestad & Skovholt, 2003). Following this research line, the present study examined the association between some personal identity factors and Professional Self-Efficacy during eTherapy in a synchronous video-based setting, by specifically investigating the relationships with Self-Esteem, General Self-Efficacy, and Insight Orientation.

Self-esteem refers to individuals’ global evaluation of themselves as persons (Rosenberg, 1965b). It is a constitutive and supportive element of one’s personal identity (Luyckx et al., 2013): individuals with higher levels of self-esteem are more inclined to consider themselves worthy and capable, whereas individuals with lower self-esteem are more likely to doubt their self-worth (see Pyszczynski et al., 2004 for a review). Consistently, self-esteem has shown negative relationships with depression and anxiety (see Sowislo & Orth, 2013, for a meta-analysis), and positive associations with well-being (Pandey et al., 2021), happiness (Dogan et al., 2013), and greater openness towards self-insight (Kwan et al., 2004). Furthermore, in the organizational field, it was related to high skill confidence (Betz & Klein, 1996), career decision self-efficacy (Choi et al., 2012), and occupational self-efficacy (Peng et al., 2021). Within psychotherapy, the relevance of Self-Esteem is also supported by evidence that therapists’ personal self-concept, in interaction with professional self-doubt, is associated with patient change and may be expressed through therapists’ in-session behavior (Nissen-Lie et al., 2017).

General Self-Efficacy refers to an element of one’s personal identity that concerns people’s beliefs about their ability to activate and use their motivations and personal resources to meet situational demands (Bandura, 1986, 1997; Berzonsky, 1992; Zhou, 2016). Individuals with high levels of self-efficacy perceive themselves as effective in overcoming difficulties to achieve their goals, and tend to persist in facing obstacles, increasing the chances of success; on the other hand, those with lower self-efficacy usually have lower aspirations and greater difficulty pursuing their goals (Feltz et al., 2008). General self-efficacy has been associated with optimism (Salanova et al., 2011) and subjective well-being (Céspedes et al., 2021), as well as with a greater aptitude for reflexivity (van Seggelen-Damen & van Dam, 2016; Gori et al., 2021a). In the professional field, individuals with higher general self-efficacy showed better performance (Lee & Ko, 2010), as well as higher commitment (Bowling et al., 2010), job satisfaction (Kondratowicz et al., 2022), and occupational self-efficacy (Betz & Klein, 1996). Among psychotherapy trainees, General Self-Efficacy has shown an association with Counseling Self-Efficacy, suggesting that broad beliefs about one’s ability to cope with demands may provide a general basis for confidence in performing therapy-specific tasks (Hahn et al., 2021).

Finally, previous evidence showed that the perception of the self is significantly associated with self-reflection and self-insight (Cowden & Meyer-Weitz, 2016). Insight is a complex construct (Castonguay & Hill, 2007) definable as a “dynamic variable that ranges from superficial consciousness to complex comprehension of elaborated emotional material” (Gori et al., 2015, p. 298). It is associated with adaptive behaviors (Gabbard, 2014) and a better quality of life (Gibbons et al., 2009). Furthermore, people with higher insight orientation also showed higher job satisfaction (Gori & Topino, 2020), job crafting (Gori et al., 2021b), and professional self-efficacy (Gori et al., 2022a). Insight Orientation may be particularly relevant to psychotherapy because self-reflection and self-awareness help clinicians distinguish their own needs and reactions from those of clients, recognize transference and countertransference processes, and make more considered clinical and ethical decisions (Gori et al., 2015; Prasko et al., 2023).

Taken together, previous research has highlighted both the relevance of these personal dimensions to therapists’ professional functioning and the close interplay between personal and professional aspects of therapist development (Alves & Gazzola, 2011; Rønnestad & Skovholt, 2003; Nissen-Lie et al., 2017; Hahn et al., 2021; Prasko et al., 2023). However, studies examining how Self-Esteem, General Self-Efficacy, and Insight Orientation are jointly associated with Professional Self-Efficacy in synchronous video-based eTherapy remain limited.

Given this theoretical and empirical framework, the present study examined the associations of Self-Esteem, General Self-Efficacy, and Insight Orientation with Therapist Self-Efficacy during synchronous video-based eTherapy in a sample of mental health professionals. To this end, a path analysis mediation model was tested, with the following hypotheses:

**H1:**  *Self-esteem and General Self-Efficacy would be significantly and positively associated with Professional Self-Efficacy;*

**H2:**  *Self-esteem and General Self-Efficacy would be significantly and positively associated with Insight Orientation;*

**H3:**  *Insight Orientation would be significantly and positively associated with Professional Self-Efficacy;*

**H4:**  *Insight Orientation would significantly mediate, either partially or fully, the associations of Self-Esteem and General Self-Efficacy with Therapist Self-Efficacy.*

Finally, previous evidence has suggested that clinical experience may be associated with therapists’ perceived professional efficacy (Wampold et al., 2017). Although evidence concerning this association in eTherapy remains limited (e.g., Gori et al., 2022a), Years of Clinical Practice was included as a covariate to examine whether the hypothesized associations remained significant after accounting for general clinical seniority.

## 2. Materials and Methods

### 2.1. Study Design, Participants, and Procedure

This study adopted a quantitative, non-experimental, cross-sectional, and correlational design. The study was conducted in Italy, and data were collected during a single recruitment wave lasting approximately 6 months through an online self-report survey administered via Google Forms. No pilot study was conducted prior to the main data collection. A sample of 290 mental health professionals was involved in this research (see Table 1). Most participants were female (n = 257, 88.6%). Participants ranged in age from 25 to 71 years (M_age = 38.25, SD = 8.40). Concerning professional qualification, 39% were psychologists and 61% were psychotherapists. Participants were recruited through a non-probability snowball sampling procedure. Eligibility criteria were being a licensed psychologist, with or without a specialization in psychotherapy, having sufficient proficiency in Italian, and providing electronic informed consent. No additional exclusion criteria were applied. The survey link was initially disseminated through the researchers’ professional contacts and through social media groups whose membership was restricted to mental health professionals. The recruitment message invited licensed psychologists to participate in a study on professional self-efficacy and encouraged them to share the survey with other colleagues who met the eligibility criteria. More detailed information about the specific aims and procedures of the research was provided on the first page of the survey.

The survey consisted of four sections. The first section provided detailed information about the study aims and procedures. The second section presented the informed consent form, and only participants who provided their consent were allowed to continue. The third section collected demographic and professional information, including age, sex, professional qualification, Years of Clinical Practice, and, for psychotherapists, Theoretical Orientation. The fourth section included the self-report measures. The survey required approximately 15 min to complete. All survey questions were mandatory; consequently, participants could not submit the questionnaire with unanswered items, and no item-level missing data were present in the final dataset. Participation was voluntary, and no financial or other incentives were offered. All procedures of this research were approved by the first author’s institutional Ethicas Committee.

### 2.2. Measures

#### 2.2.1. Therapist Self-Efficacy Scale (T-SES)

The *Therapist Self-Efficacy Scale* (T-SES; Gori et al., 2022a) is a self-report measure designed to assess mental health therapists’ professional self-efficacy. The T-SES consists of 21 items rated on a 5-point Likert scale, from 1 (*not at all*) to 5 (*a great deal*). The scale was originally validated to assess clinicians’ self-confidence during eTherapy (Gori et al., 2022a). The original Italian validation supported a one-factor structure explaining 70% of the variance and showed excellent internal consistency (α = 0.93; ω = 0.94). Although the items cover six clinically relevant content areas (communicative effectiveness, clinical competence, intrapsychic competence, relational competence, affect regulation, and diagnostic skills), these areas do not constitute separate subscales; therefore, only the total score, ranging from 21 to 105, was used (Gori et al., 2022a). In the present research, the total score of the original (Italian) version was used, and it showed excellent internal consistency (Cronbach’s α value of 0.97).

#### 2.2.2. General Self-Efficacy Scale (GSE)

The *General Self-Efficacy Scale* (GSE; Schwarzer & Jerusalem, 1995) is a self-report measure designed to assess general self-efficacy. The GSE consists of 10 items rated on a 4-point Likert scale, from 1 (*not at all true for me*) to 4 (*very true for me*). Cross-national psychometric analyses supported the unidimensional structure of the scale, with internal consistency values of α = 0.81 for the German version and α = 0.79 for the Italian adaptation (Scholz et al., 2002). The total score ranges from 10 to 40, and no subscale scores were calculated. In the present research, the total score of the Italian version (Sibilia et al., 1995) was used, and it showed excellent internal consistency (Cronbach’s α value of 0.90).

#### 2.2.3. Rosenberg Self-Esteem Scale (RSES)

The *Rosenberg Self-Esteem Scale* (RSES; Rosenberg, 1965a) is a self-report measure designed to assess global self-esteem. The RSES consists of 10 items rated on a 4-point Likert scale, from 0 (*strongly disagree*) to 3 (*strongly agree*). The original version showed a coefficient of reproducibility of 0.92 (Rosenberg, 1965a, 1965b), whereas the Italian validation showed good internal consistency (α = 0.84) and test–retest reliability (r = 0.76; Prezza et al., 1997). The RSES provides a global Self-Esteem score ranging from 0 to 30; therefore, no subscale scores were calculated. In the present research, the total score of the Italian version (Prezza et al., 1997) was used, and it showed good internal consistency (Cronbach’s α value of 0.81).

#### 2.2.4. Insight Orientation Scale (IOS)

The *Insight Orientation Scale* (IOS; Gori et al., 2015, 2022b) is a self-report measure designed to assess the levels of insight. The IOS consists of 7 items scored on a 5-point Likert scale, from 1 (*not at all*) to 5 (*a great deal*). The original validation showed satisfactory internal consistency (α = 0.77) and evidence of concurrent and discriminant validity, while a subsequent Item Response Theory analysis further supported its psychometric stability and measurement precision (Gori et al., 2015, 2022b). The total score ranges from 7 to 35, and the seven components assessed by the items do not constitute separate subscales. In the present research, the total score of the original (Italian) version was used, and it showed good internal consistency (Cronbach’s α value of 0.86).

### 2.3. Data Analysis

All statistical analyses were performed using SPSS 21.0 (v. 21.0; IBM, New York, NY, USA) and AMOS 24.0 (v. 24.0; IBM, New York, NY, USA). Descriptive statistics were calculated. Partial correlations controlling for Years of Clinical Practice were calculated to examine associations among the study variables. Then, the hypothesized model was tested by implementing a path analysis (Bollen & Long, 1993), to investigate the mediation of Insight Orientation in the relationship of Self-Esteem and General Self-Efficacy with Therapist Self-Efficacy during eTherapy, also controlling for Years of Clinical Practice. The following goodness-of-fit indicators were used to assess the statistical fit of the model: the model Chi-square (*χ*^2^), indicating a good model fit when *p* > 0.05 (Hooper et al., 2008); the normed-fit index (NFI), Tucker–Lewis index (TLI), and Comparative Fit Index (CFI) indicating a reasonable fit for values above 0.90 (Byrne, 1994; Kline, 2015; Hu & Bentler, 1999); the root mean square error of approximation (RMSEA) and the standardized root mean square residual (SRMR), indicating a reasonable fit for values below 0.08 (Hooper et al., 2008; Marsh & Hocevar, 1985). The *R*^2^ index was also explored, considering values *<* 0.02 as indicative of very weak effect, values between 0.02 and 0.12 as indicative of a weak effect, values between 0.13 and 0.26 as indicative of a moderate effect, values *>* 0.26 as indicative of a substantial effect (Cohen, 1988). The bootstrapping technique (5000 bootstrapped samples with 95% bias-corrected bootstrap confidence intervals) was performed to support the statistical stability of the model (Preacher & Hayes, 2008). Finally, a post hoc power analysis was conducted using G*Power version 3.1.9.7 (Faul et al., 2009), based on a fixed-effects linear multiple regression model testing the deviation of R^2^ from zero. In accordance with Cohen’s (1988) conventional criterion, statistical power values of 0.80 or higher were considered adequate.

## 3. Results

The largest proportion of participants (32%) declared more than 10 years of clinical practice (see Table 1). Concerning the Psychotherapists’ Theoretical Orientation, 13% of the sample were integrated, 11% systemic, 10% cognitive behavioral, 10% psychodynamic, 6% cognitive, 6% humanistic, and 5% psychoanalytic (see Table 1).

Correlation analysis showed significant and positive relationships between the observed variables (see Table 2). Therapist Self-Efficacy during eTherapy was significantly and positively associated with General Self-Efficacy (*r* = 0.33, *p* < 0.01), Self-Esteem (*r* = 0.29, *p* < 0.01), and Insight Orientation (*r* = 0.51, *p* < 0.01).

**Figure 1:**
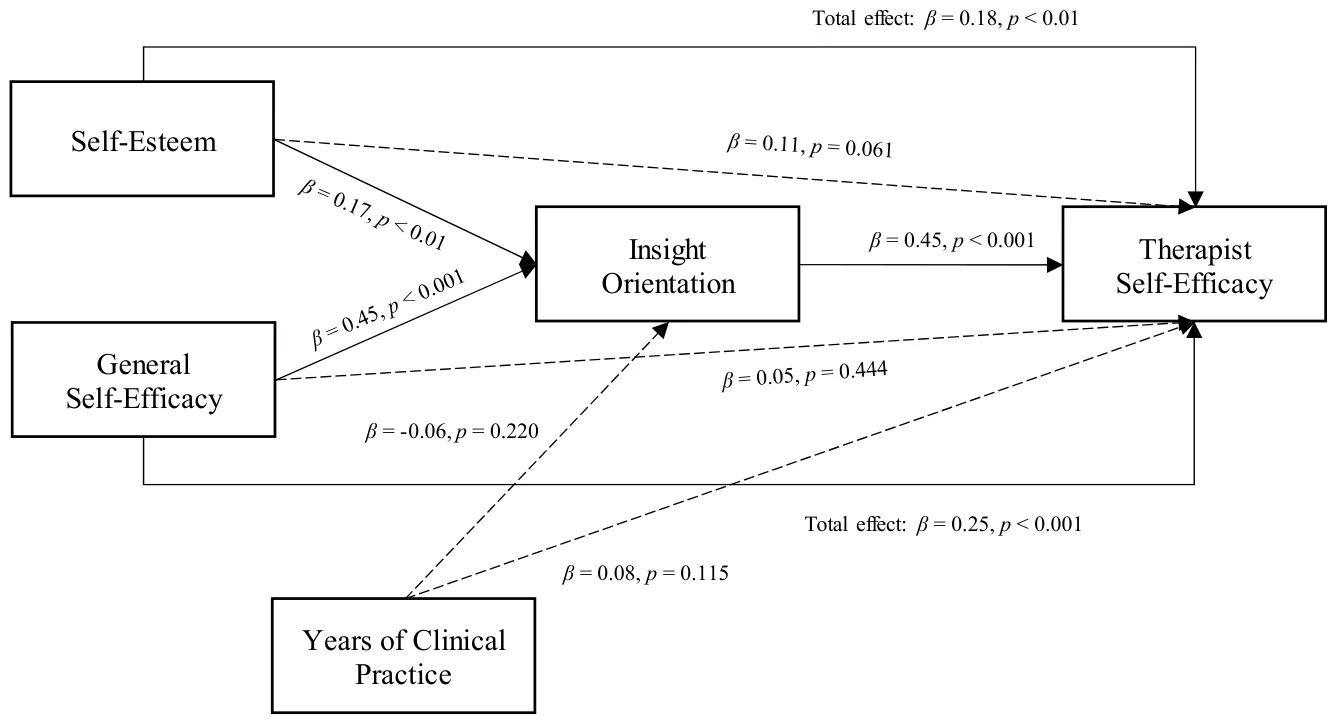
Path analysis model examining the mediation of Insight Orientation in the associations of Self-Esteem and General Self-Efficacy with Therapist Self-Efficacy, controlling for Years of Clinical Practice.

Concerning the path analysis, the hypothesized path model showed a satisfactory overall fit: *χ*^2^ (2) = 5.721 (*p* = 0.057), NFI = 0.979; TLI = 0.927, CFI = 0.985, RMSEA = 0.080, SRMR = 0.042 (see Figure 1).

Specifically, Self-Esteem and General Self-Efficacy showed a significant total effect in their relationship with Therapist Self-Efficacy during eTherapy (**H1**; *β* = 0.18, *p* < 0.01 and *β* = 0.25, *p* < 0.001, respectively). Furthermore, both Self-Esteem and General Self-Efficacy were significantly and positively associated with Insight orientation (**H2**; *β* = 0.17, *p* < 0.01 and *β* = 0.45, *p* < 0.001, respectively), which in turn showed a significant relationship with Therapist Self-Efficacy during eTherapy (**H3**; *β* = 0.45, *p* < 0.001). Concerning the covariate, no significant effects have been highlighted between Years of Clinical Practice and Insight Orientation (*p* = 0.220) and Therapist Self-Efficacy during eTherapy (*p* = 0.115). Finally, when included in the model, Insight orientation fully mediated the effect of Self-Esteem and General Self-Efficacy on Therapist Self-Efficacy during eTherapy, determining non-significant direct effects (**H4**; see Figure 1 and Table 3).

The model explained 28% of the total variance, indicating a substantial effect (*R*^2^ > 0.26).

The post hoc power analysis yielded a value exceeding the conventional adequacy criterion of 0.80, indicating adequate statistical sensitivity to detect the observed overall model effect.

The bootstrapping technique supported the statistical stability of the model (see Table 3).

## 4. Discussion

Technology and the Internet have become increasingly integrated into mental health practice. Evidence indicates that eTherapy can be effective across different therapeutic approaches, diagnostic and assessment contexts, and populations, with treatment outcomes comparable to those of in-person interventions (Barnett et al., 2021; Bashshur et al., 2016; Hilty et al., 2013; Lin et al., 2022). Because therapeutic outcomes are also associated with clinicians’ characteristics and perceptions (the so-called therapist effect; Barkham et al., 2017), therapists’ confidence in their ability to facilitate therapeutic processes through synchronous video-based care warrants specific attention. Accordingly, the present research aimed at exploring the interaction of different personal characteristics in influencing Therapist Self-Efficacy during eTherapy in a synchronous video-based setting by focusing on Self-Esteem, General Self-Efficacy, and Insight Orientation.

Overall, the findings suggest that general evaluations of personal worth and capability and the tendency to reflect on and understand one’s internal experiences represent distinct but interrelated dimensions associated with clinicians’ confidence in providing online psychological interventions. Self-Esteem and General Self-Efficacy showed significant and positive total effects in their relationships with Professional Self-Efficacy, supporting the first hypothesis (**H1**). This was in line with previous research. Indeed, higher levels of self-esteem have been associated with higher job satisfaction (Reilly et al., 2014), lower risk of burnout (Molero Jurado et al., 2018), and better self-conception as a professional (Glotova & Wilhelm, 2014). In parallel, individuals with greater self-efficacy presented a greater perception of professional competence (Yao et al., 2021), higher resilience scores (Orkaizagirre-Gómara et al., 2020) and professional commitment (Cheng et al., 2021), as well as lower perceived stress (Orkaizagirre-Gómara et al., 2020). Taken together, the present findings and previous evidence suggest that general evaluations of personal worth and capability may also be reflected in the way clinicians evaluate their ability to manage the specific demands of online therapeutic practice. These broad personal resources may be particularly relevant when therapists must sustain confidence while managing technology-mediated communication, possible technical disruptions, and reduced access to some non-verbal and contextual cues (Simpson & Reid, 2014; Stoll et al., 2020).

Self-Esteem and General Self-Efficacy were also significantly and positively related to Insight Orientation, confirming the second hypothesis (**H2**). Consistently, previous evidence showed a significant and positive relationship between self-esteem and self-insight (Kwan et al., 2004), as well as the significant predictive role of general self-efficacy on reflection (van Seggelen-Damen & van Dam, 2016), one of the core constitutive elements of insight orientation (Castonguay & Hill, 2007; Gori et al., 2015). In turn, Insight Orientation was significantly and positively associated with Professional Self-Efficacy, in line with the third hypothesis (**H3**). Reflective thinking and self-awareness have been identified as fundamental competencies for mental health professionals by the British Psychological Society (2006). The American Psychological Association (2012) also included these components in its definition of “Professionalism”, which encompasses reflective practice, self-evaluation, and self-care. In the specific context of eTherapy, Insight Orientation may be relevant because professional confidence does not depend solely on technical knowledge. Online clinical practice may also require therapists to monitor their own reactions, recognize personal strengths and limitations, and adapt established therapeutic skills to a technologically mediated relational setting.

Consistently, results also showed that Insight Orientation fully mediated the associations of Self-Esteem and General Self-Efficacy with Therapist Self-Efficacy, confirming the final hypothesis (**H4**). This pattern can be interpreted in light of the literature highlighting the relevance of personal factors to professional confidence (Holland et al., 2012; Gori et al., 2023). In this regard, Nuttman-Schwartz (2017) argued that individuals simultaneously inhabit multiple identities that, although distinguishable, are deeply intertwined (Wijeyesinghe & Jones, 2014). Accordingly, the present findings are consistent with the view that personal identity-related dimensions are associated with professional identity, understood as the meanings individuals attribute to themselves within their professional context (Wiles, 2013). Conceptually, Self-Esteem and General Self-Efficacy reflect broad evaluations of personal worth and capability, whereas Insight Orientation involves reflective understanding of internal experiences (Gori et al., 2015). It may therefore represent the process through which these general personal resources are associated with a more differentiated appraisal of therapists’ professional strengths and limitations, consistently with models emphasizing reflection in therapist development (Rønnestad & Skovholt, 2003; van Seggelen-Damen & van Dam, 2016; Gori et al., 2022a). In synchronous video-based eTherapy, this reflective dimension may be especially relevant because therapists must evaluate their ability to adapt communication, relational attunement, and established clinical skills to the online medium (Simpson & Reid, 2014; Stoll et al., 2020).

Finally, Years of Clinical Practice was included as a covariate and was not significantly associated with either Insight Orientation or Therapist Self-Efficacy during eTherapy. This result is consistent with previous evidence (Gori et al., 2022a) and suggests that confidence in online clinical practice may not be directly related to the duration of face-to-face clinical experience. One possible interpretation is that eTherapy presents specific technological, communicative, and relational demands that differ from those encountered in traditional in-person practice. The technologically mediated setting may also reduce some of the advantages ordinarily associated with clinical seniority, as established therapeutic skills and routines must be adapted to different channels of communication and interaction. These findings therefore suggest the potential value of training specifically focused on online clinical practice, in line with previous evidence linking education and training to therapist self-efficacy (Meyer, 2015; Poletti et al., 2020; Reese et al., 2009). Familiarity with video-based platforms, previous eTherapy practice, specific telepsychology training, and perceived digital competence may be more relevant than the overall number of years spent in face-to-face clinical work. Adaptation to eTherapy may also be associated more closely with flexibility and attitudes toward technology than with professional seniority alone. Although younger clinicians may be more familiar with digital communication, greater age or clinical experience does not necessarily imply lower adaptability; therefore, these possibilities require direct empirical examination. Research on psychologists’ digital competence has highlighted considerable variability across digital tasks and identified clinicians’ skills, technical conditions, workplace support, and training as relevant dimensions of digital practice (Dobson et al., 2022). Future studies should therefore distinguish general clinical seniority from eTherapy-specific experience and directly assess digital competence, previous training, frequency of online sessions, attitudes toward technology, flexibility in adapting clinical practices, and familiarity with digital platforms.

The model explained 28% of the variance in Therapist Self-Efficacy, suggesting that the personal dimensions examined account for a meaningful, although necessarily partial, proportion of clinicians’ confidence in providing eTherapy. At the same time, alternative explanations and potential contextual confounders should be considered. Professional self-efficacy may also be associated with factors more specific to digital practice, including prior exposure to online therapy, attitudes toward technology, eTherapy-related preparation, digital competence, targeted training, and workplace or technical support (Chen et al., 2023; Dobson et al., 2022). These variables may further shape clinicians’ familiarity with digital platforms and their confidence in managing the technical and relational demands of online practice, and may therefore have contributed to the observed pattern of associations. Thus, the present model highlights a relevant set of personal correlates within the broader, multifactorial nature of Therapist Self-Efficacy.

The present study has some limitations that should be addressed. First, the cross-sectional design does not allow the temporal ordering or causal direction of the observed associations to be established. Alternative temporal sequences and reciprocal relationships are also plausible. Future longitudinal studies, particularly those including repeated assessments and cross-lagged analyses, could clarify the direction, temporal development, and possible reciprocal nature of these associations.

Furthermore, the sample was predominantly female, which may limit the generalizability of the findings to male professionals. This marked gender imbalance also prevented an in-depth examination of potential gender-related differences. Information concerning participants’ ethnicity, cultural background, and geographic distribution was also not collected, limiting the assessment of the sample’s sociocultural and geographic representativeness. Future studies could strengthen the generalizability of the present findings by including more gender-balanced and socioculturally and geographically diverse samples.

In addition, the online snowball sampling procedure may have preferentially reached professionals who were more familiar with digital technologies or more interested in eTherapy. Although this recruitment strategy enabled access to a relevant professional population, it may have resulted in a greater representation of clinicians who felt more comfortable with online practice. Future research could complement online recruitment with broader professional channels to include clinicians with different levels of digital experience and confidence in eTherapy.

Finally, all key variables were assessed through validated self-report measures. Although these instruments provided reliable information on participants’ perceptions, the use of a single assessment method may be associated with common method variance and response biases. Future studies could integrate self-report measures with interviews, supervisor evaluations, behavioral indicators, or client-reported outcomes to provide a more comprehensive assessment.

## 5. Conclusions

Among the factors associated with effective therapeutic practice, previous evidence has highlighted the relevance of mental health professionals’ confidence in their personal and professional resources and in their ability to apply them within clinical work (Hatcher et al., 2013; Knapp et al., 2017). In light of this, and given the growing integration of the Internet into mental health practice, the present study focused on factors associated with Professional Self-Efficacy during synchronous video-based eTherapy. Specifically, it examined its associations with Self-Esteem, General Self-Efficacy, and Insight Orientation, while controlling for Years of Clinical Practice as a covariate. Results showed significant positive total associations of Self-Esteem and General Self-Efficacy with Therapist Self-Efficacy, both of which were fully mediated by Insight Orientation. This interpretation is consistent with Gori et al.’s (2022a) conceptualization of Therapist Self-Efficacy as therapists’ awareness and appraisal of their ability to facilitate therapeutic processes and promote change across clinical settings. Finally, Years of Clinical Practice was not significantly associated with Therapist Self-Efficacy, suggesting that general clinical seniority may not be directly related to confidence in providing online therapy. These findings may have relevant practical implications, pointing to the potential value of specific eTherapy training for mental health professionals. Such programs could address the technical and ethical management of online practice, including the appropriate use of digital platforms, confidentiality, data protection, informed consent, and the management of technical disruptions and emergency situations. They could also focus on adapting therapeutic communication, assessment procedures, alliance-building, and the interpretation of verbal and non-verbal cues to synchronous video-based settings. Finally, reflective supervision could support clinicians in examining their emotional reactions, perceived strengths and limitations, and confidence in managing the specific relational and organizational demands of online therapy.

## Figures and Tables

**Table 1:** Demographic and professional characteristics of the sample (N = 290).

Characteristics		M ± SD	n	%
Age		38.25 ± 8.40		
Sex	Females		257	88.6
	Males		33	11.4
Professional Qualification	Psychologists		112	38.6
	Psychotherapists		178	61.4
Years of Clinical Practice	Less than a year		22	7.6
	1–2 years		34	11.7
	2–5 years		63	21.7
	5–10 years		78	26.9
	More than 10 years		93	32.1
Theoretical Orientation (Psychotherapists only)	Psychoanalytic		15	5.2
	Psychodynamic		28	9.7
	Cognitive		18	6.2
	Cognitive behavioral		30	10.3
	Humanistic		17	5.9
	Integrated		38	13.1
	Systemic		32	11.0

**Table 2:** Partial correlations among the study variables, controlling for Years of Clinical Practice.

	General Self-Efficacy	Self-Esteem	Insight Orientation	Therapist Self-Efficacy
General Self-Efficacy	1			
Self-Esteem	0.44	1		
Insight Orientation	0.52	0.37	1	
Therapist Self-Efficacy	0.33	0.29	0.51	1

***Note:*** All correlations were significant at *p* < 0.01.

**Table 3:** Coefficients of the path analysis mediation model.

	Estimate	SE	*p*	BootLLCI	BootULCI
*Total effects*					
Self-Esteem → Therapist Self-Efficacy	0.183	0.062	**<0.01**	0.061	0.365
General Self-Efficacy → Therapist Self-Efficacy	0.247	0.060	**<0.001**	0.127	0.364
*Direct effects*					
Self-Esteem → Therapist Self-Efficacy	0.107	0.059	0.083	−0.015	0.216
General Self-Efficacy → Therapist Self-Efficacy	0.047	0.058	0.389	−0.063	0.165
*Indirect effects*					
Self-Esteem → Insight Orientation → Therapist Self-Efficacy	0.077	0.032	**<0.001**	0.034	0.133
General Self-Efficacy → Insight Orientation → Therapist Self-Efficacy	0.200	0.033	**<0.001**	0.133	0.282

***Note:*** Bold values indicate significant *p* values.

## Data Availability

Data presented in this study are available on request from the corresponding author.
